# Case Report: Lung transplant recipient with bullous pemphigoid and antibody-mediated rejection: an overlapping immunologic phenomenon?

**DOI:** 10.3389/frtra.2026.1773797

**Published:** 2026-04-15

**Authors:** Sandrine Hanna, Daniel Gutteridge, Christiane Machado, Mrunal G. Patel, David W. Roe, Nichole A. Smith, Biplab Saha

**Affiliations:** 1 Pulmonary, Critical Care & Lung Transplant, Indiana University Health University Hospital, Indianapolis, IN, United States; 2 Department of Dermatology, Indiana University Hospital Indiana, Indianapolis, IN, United States

**Keywords:** antibody-mediated rejection, blistering skin disease, bullous pemphigoid, donor specific antibodies, lung transplantation

## Abstract

We present what is, to our knowledge, the first reported case of concurrent bullous pemphigoid (BP) and antibody-mediated rejection (AMR) in a lung transplant patient. Similar associations have been described in other solid organ transplant settings, most notably in renal transplantation, where explantation of the allograft has frequently been followed by resolution of the skin disease. These observations suggest a broader potential connection between autoimmune blistering disorders and allograft rejection mechanisms, especially as emerging evidence highlights the role of non–HLA antibodies. A comprehensive review of previously reported cases of BP in solid organ transplant recipients is therefore warranted to place this case in context and to assess its significance within the existing literature.

## Introduction

Bullous pemphigoid (BP) is the most common autoimmune blistering skin disease in the Western Hemisphere (1). It is characterized by the presence of antibodies to hemidesmosome proteins, which clinically manifests as the separation of the dermis and epidermis (2). Its association with rejection in solid organ transplant (SOT) is rare and has been mostly described in renal and Hematopoietic Stem Cell Transplant (HSCT) recipients. We report a case of concurrent onset of antibody-mediated rejection (AMR) in a lung transplant patient with BP. Importantly, exacerbations of BP temporally preceded increases in antibody titers and subsequent deterioration in lung function, suggesting a potential immunological connection between the two conditions.

## Case description

**Figure 1:**
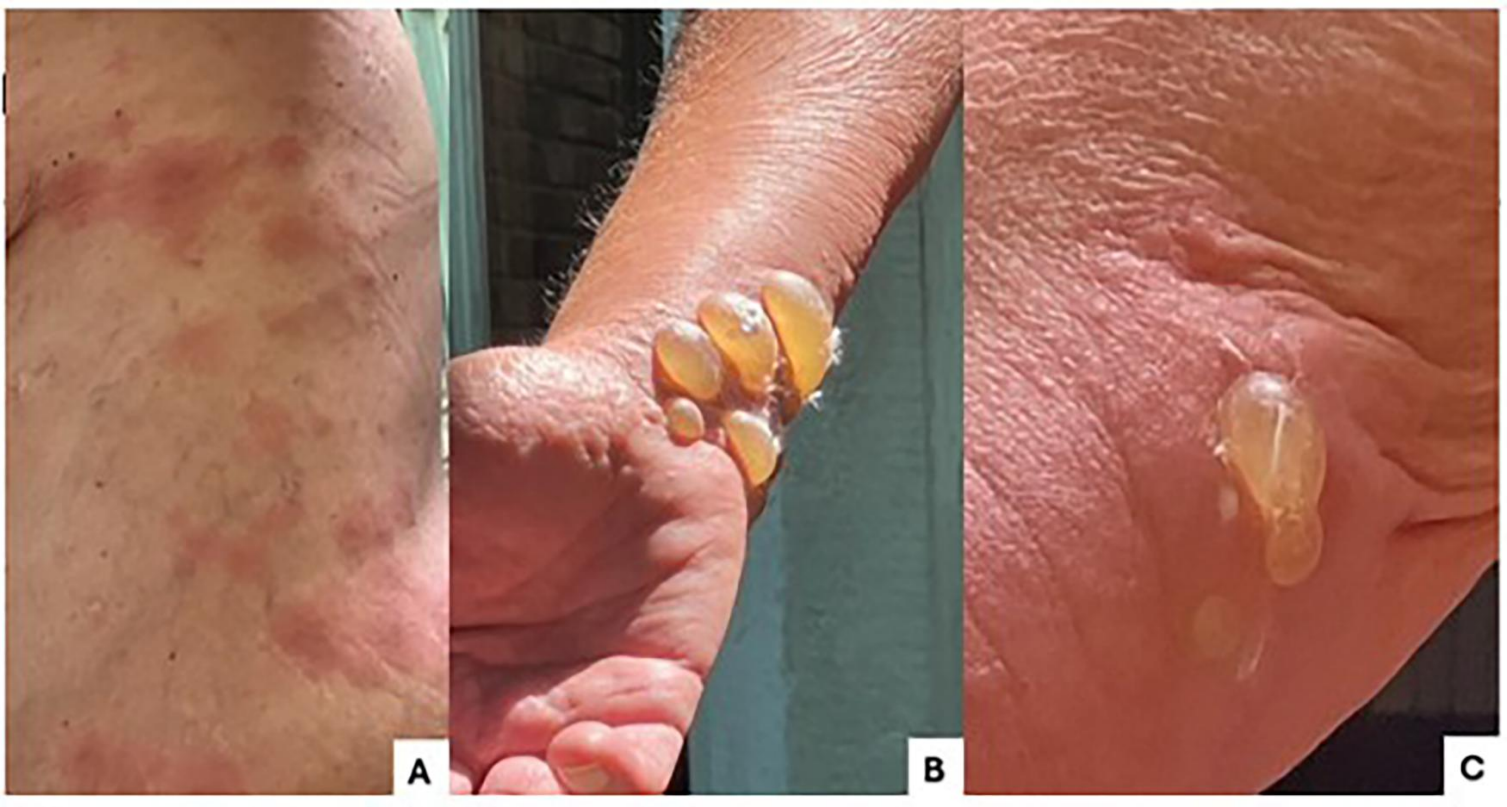
Bullous pemphigoid rash on torso **(A)**, Tense bullae formation **(B,C)**.

The patient is a 71-year-old man with a history of chronic obstructive pulmonary disease who underwent bilateral lung transplantation. No other relevant medical, social, or family history was reported. Approximately two years following the transplant, the patient developed a pruritic rash followed by the formation of large tense bullae, which initially appeared on the hands and later extended to the thighs and torso (Figure 1). The condition was initially presumed to be contact dermatitis, as he reported prior contact with poison sumac. He was treated with steroids without any clinical improvement and with further extension of the rash area. He was referred to dermatology, and a diagnosis of bullous pemphigoid was confirmed by punch biopsies, Direct Immunofluorescence (DIF), and an elevated titer of the IgG BP 180 antibodies by ELISA test. His prednisone dose was increased, and adjunctive topical corticosteroids were administered. Niacinamide and a course of doxycycline were added to his treatment plan.

**Figure 2:**
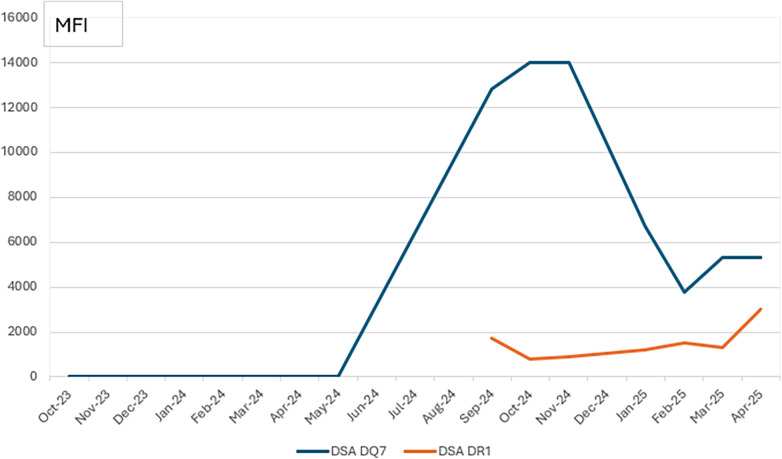
DeNovo DSA with MFI titers.

During the same period, he also reported progressive exertional dyspnea. Chest imaging, including a chest computed tomography (CT) was unremarkable. His pulmonary function testing revealed a 13% decline in FEV₁ (Forced expiratory volume in 1 s) from his personal best. His *de novo* donor-specific antibodies (DSA) were positive for DQ7 and DR1, with a mean fluorescence index (MFI) of 12,800 and 1,700, respectively, as determined by the Single-Antigen Bead Luminex assay (SAB) (Figure 2). The patient underwent bronchoscopy with transbronchial biopsies, but the samples were inadequate for histopathologic analysis. The bronchoalveolar lavage was negative for any microbiologic pathogen. Given his denovo DSA, decline in PFT, and absence of any other etiology for lung function decline, he was diagnosed with possible clinical AMR. At the time of AMR development, the patient's immunosuppressive regimen included tacrolimus, sirolimus, prednisone, and mycophenolate.

**Figure 3:**
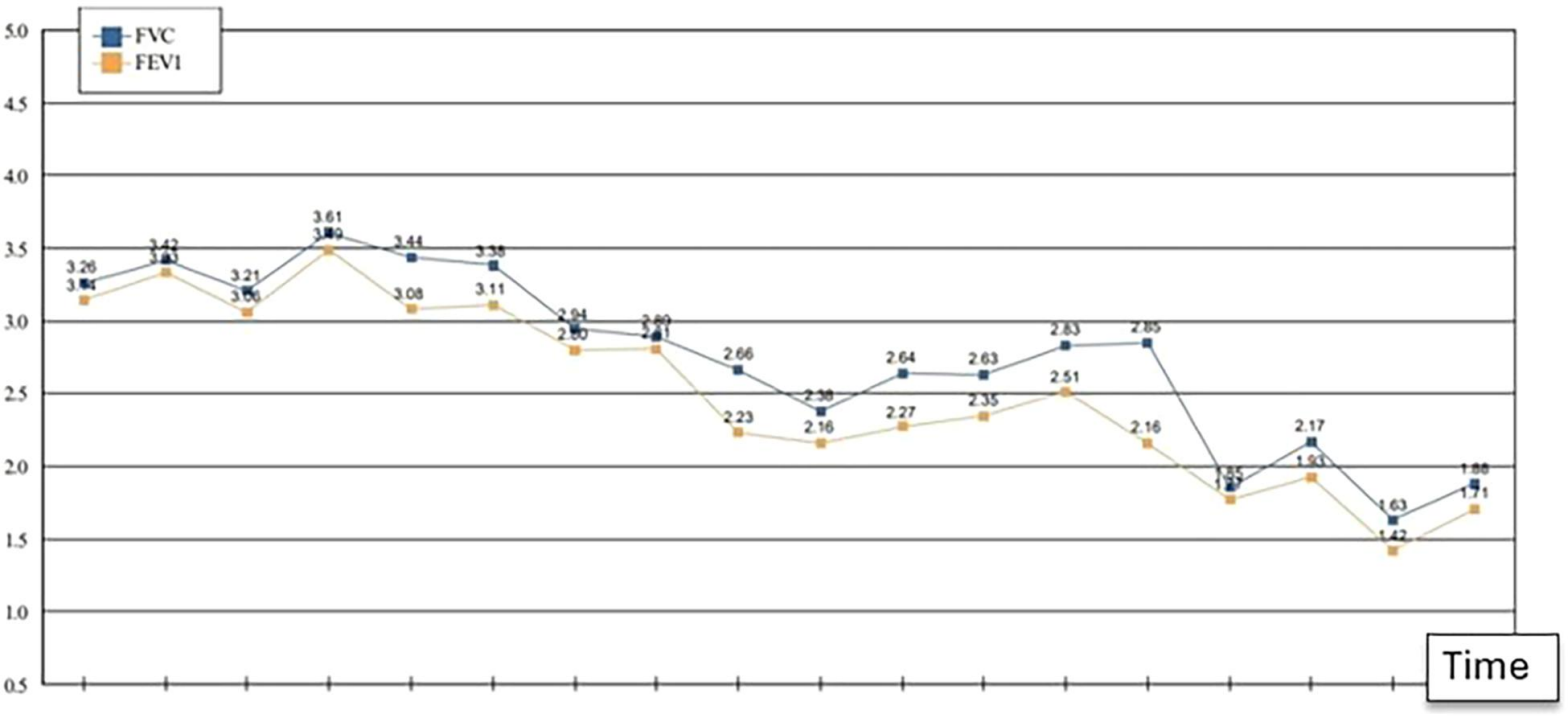
Declining pulmonary function. Forced Expiratory Capacity (FEV1); Forced Vital Capacity (FVC) in Liters (L).

**Figure 4:**
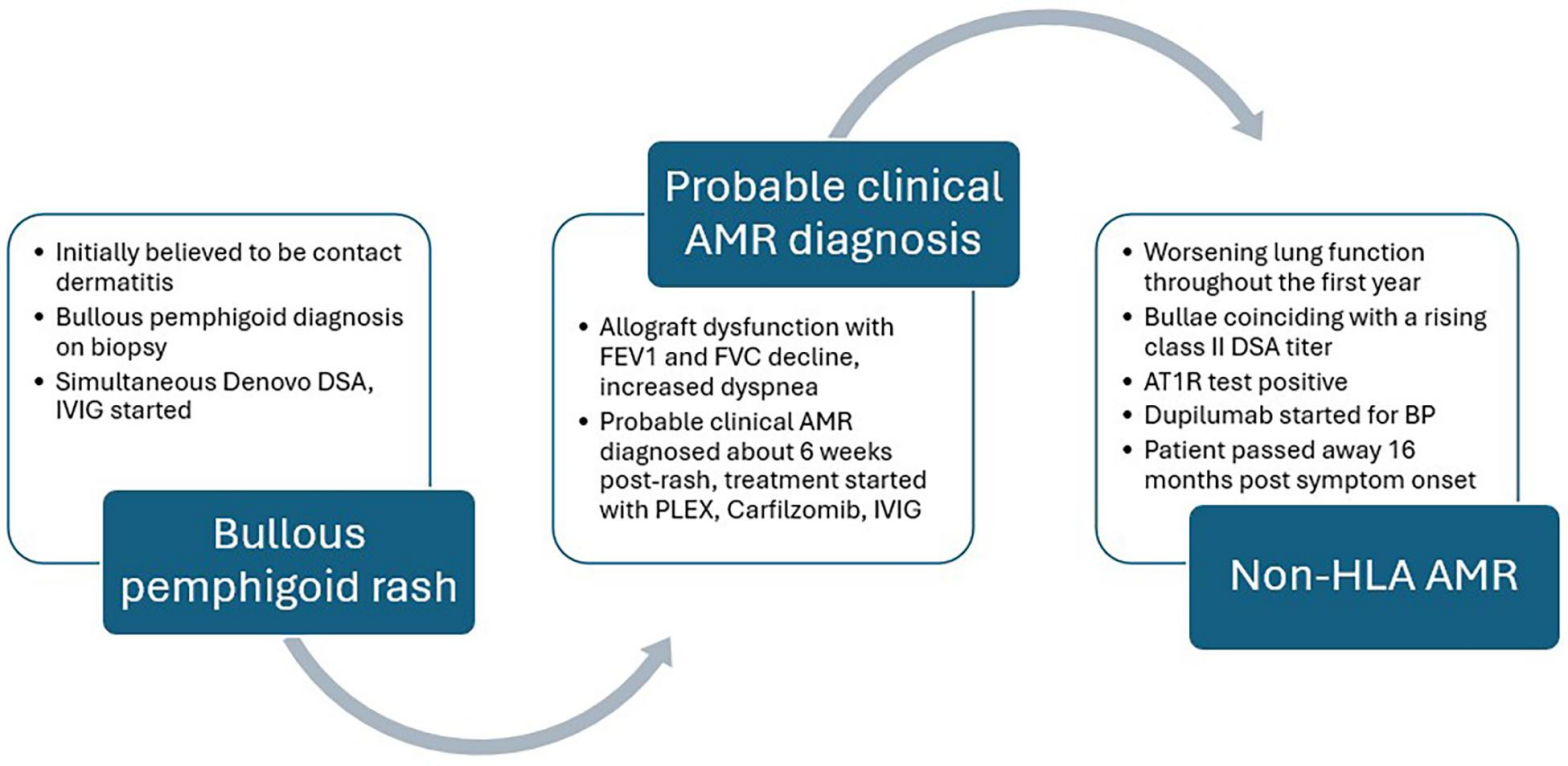
Timeline of events. FEV(1), forced expiratory capacity (1); FVC, forced vital capacity; DSA, donor-specific antibodies; IVIG, intravenous immunoglobulin; AMR, antibody-mediated rejection; PLEX, plasmapheresis; BP, bullous pemphigoid; Anti-angiotensin II type-1 receptor antibody, A1TR.

The patient was hospitalized for the protocolized treatment of AMR, which included plasmapheresis, Carfilzomib, and intravenous immune globulins (IVIG). Unfortunately, he experienced an ongoing decline in his respiratory status over the next year (Figure 3) and developed pleuroparenchymal fibrotic changes with traction bronchiectasis on CT imaging. During this time, he experienced frequent flares of his BP, which appeared to parallel rising donor-specific antibody (DSA) titers and continued decline in pulmonary function tests. He also tested positive for anti-angiotensin II type-1 receptor antibody (AT1R test), indicating the presence of non-HLA type antibodies. Dupilumab was added for the treatment of his bullous pemphigoid based on published case reports in the renal transplant literature, with subsequent improvement in his itching and rash. The patient passed away approximately 16 months after being diagnosed with AMR. A timeline of major clinical events is represented in Figure 4.

## Discussion

BP disease hallmarks include intense pruritus, urticarial or eczematous lesions, and tense bullae on the trunk and flexural extremities. In certain cases, pruritus may precede blisters by weeks to months. Its diagnosis requires at least two punch biopsies—one for hematoxylin-eosin staining and one for direct immunofluorescence (DIF). This is often complemented by serological tests such as enzyme-linked immunosorbent assays to detect circulating IgG autoantibodies to hemidesmosomal proteins BP180 (also known as type XVII collagen) and BP230, and indirect immunofluorescence on basement membrane zone split skin to screen for basement membrane zone-associated autoantibodies (3). BP can significantly impact patients' quality of life and is associated with increased mortality and morbidity; hence, routine disease monitoring is recommended. This assessment is conducted using clinician-reported outcomes (e.g., Autoimmune Bullous Skin Disorder Intensity Score), patient-reported outcome measures (e.g., Skindex, Bullous Pemphigoid Disease Area Index, Dermatology Life Quality Index), and serological analyses (3).

Familial aggregation of BP is not common, suggesting that it is not a pure genetic disease. However, numerous genomic regions, particularly within the HLA class II locus, have been implicated in bullous pemphigoid susceptibility based on findings from targeted sequencing studies (2). Studies in different ethnic populations demonstrated that HLA-DQ and DR were the strongest predictors of genetic risk (particularly HLA-DQB1*0301 alleles in several ethnic populations) (2). The underlying mechanism may involve the HLA protein encoded by DQB1*03:01, which can bind to multiple T-cell epitopes within both BP230 and BP180 (4).

Several groups of medications have been associated with the development of BP. Particularly, aldosterone antagonists, immune checkpoint inhibitors (ICIs), anti-tumor necrosis factor (TNF)-*α* monoclonal antibodies, dipeptidyl peptidase-4 inhibitors (DPP-4 inhibitors, also known as “gliptins”), anticholinergics, diuretics, antibiotics, and dopaminergic agents. Other reported predisposing risk factors include external skin trauma, including physical (i.e, surgical procedures), thermal, ultraviolet (UV) light injuries, burns, and radiotherapy. It has also been reported with the use of certain vaccines (influenza, tetanus, herpes), although the association is not clear. infections (notably human herpes viruses, HHV) (5). Transplantation, both solid organ (SOT) and hematopoietic stem cell transplant (HSCT), has also been linked to BP.

Transplantation is one of the reported factors associated with BP, especially in the renal transplant population (5). In the renal transplant literature, most cases were associated with chronic rejection and treated with steroids and immune suppression augmentation, and sometimes nephrectomy. It is hypothesized that the mechanism involves either the production of autoantibodies that cross-react between the skin and the transplanted organs, or through immune suppression and a decrease in the number of circulating regulatory T cells (Tregs) (6). Several case reports documented the resolution of BP following the removal of the transplanted organ (7–9)

While a systematic review has been conducted about the occurrence of BP in Allogeneic Hematopoietic Stem Cell Transplant (HSCT), along with several studies in the renal transplant population (6, 10, 11), none have been reported to date in the general SOT population (12). We include cases of bullous pemphigoid in the solid organ transplant (SOT) population beyond renal transplant recipients.

A comprehensive literature search was performed in PubMed and Embase through September 2025 using the keywords “bullous pemphigoid”, 'solid organ transplantation', and “autoimmune blistering disease”. All case reports and series in SOT were considered eligible except for renal cases and stem cell transplants. Two cases of bullous pemphigoid (BP) have been reported following liver transplantation, and one case has been documented after bilateral hand transplantation (Table 1). To date, there have been no reported cases of BP in recipients of lung or heart transplants.

**Table 1:** Summary of BP cases in SOT.

Case	Author, year	Organ type	Time from Tx to BP onset (months/ years)	Rejection	Age at BP	IS	Diagnosis
1 (16)	Rashid, 2025	Liver	29 years	Unknown	70	CYA	Bx/DIF
2 (17)	Kerkar, 2006	Liver	10 months	No, but liver dysfunction	∼18 months	FK/ Pred	Bx/DIF
3 (18)	Weissenbacher	Bilateral hand	11 years	No, but preceded by radial fracture surgery	57	SRL/ Pred	Bx/DIF

CYA, Cyclosporine; IS, Immune suppression; Tx, Transplant; FK, Tacrolimus, Pred, prednisone; Bx, Biopsy; DIF, Direct immunofluorescence.

In a large-scale retrospective cohort study, Kasperkiewicz et al. evaluated the risk of BP in transplant recipients. Their findings indicated a slightly elevated, though still low, incidence of BP (<0.05%) among transplant patients, with the increased risk primarily observed in renal and bone marrow transplant cohorts. Particularly, no increased risk was identified in recipients of liver, lung, or heart transplants (13). Although a definitive association between antibody-mediated rejection (AMR) in renal allografts and the development of BP has not been clearly established in the literature, immune cross-reactivity between the skin and kidney has been proposed, primarily supported by reports linking glomerulonephritis and dermatologic manifestations (6). The immunologic basis of this phenomenon is further suggested by the resolution of BP following allograft explantation. While such an association has not been previously reported in lung transplant recipients, we describe the first documented case of concurrent AMR and BP, suggesting a potential role of circulating autoantibodies in the shared pathophysiology. Notably, an increase in pruritus was observed to precede a rise in autoantibody mean fluorescence intensity (MFI), further supporting this association.

Patients with BP develop circulating autoantibodies targeting the cutaneous basement membrane zone. This leads to the disruption of the dermal–epidermal junction (DEJ) through complement activation, inflammatory cell recruitment, the liberation of proteolytic enzymes, and the direct adhesion of antibodies. The humoral immune response in BP is typically polyclonal, directed against multiple epitopes of BP-associated antigens. Notably, the majority of autoantibodies recognize the immunodominant region of the BP180 antigen, specifically the NC16A domain (14). The humoral nature of BP is now well established, directly linking the role of autoantibodies to the development of tissue injury. The serum levels of autoantibodies to BP 180 correlate with disease severity.

This observation raises the question of whether antibody-mediated rejection (AMR) in solid organ transplantation may involve antibodies directed against components of the dermal–epidermal junction (DEJ). Several antibodies implicated in AMR are non-HLA antibodies, which have been recognized as pathogenic contributors to the disease. These antibodies are typically expressed on various cell types, including fibroblasts, endothelial cells, and epithelial cells. Examples include antibodies targeting the angiotensin II type 1 receptor (AT1R), vimentin, myosin, collagen V, and the endothelin type A receptor (ETAR). Among these, AT1R antibodies are well established in the context of kidney and heart allograft rejection. Interestingly, collagen V, a minor component of collagen in the lungs, is normally sequestered but becomes exposed following recurrent allograft injury. Similarly, K-alpha 1 tubulin, an epithelial surface gap junction cytoskeletal protein, may also become accessible under such conditions, contributing to the immune response (15). Given the current limitations in testing for all non-HLA antibodies, we hypothesize that such antibodies may play a role in the pathophysiology underlying the coexistence of AMR and BP.

This case highlights the importance of recognizing cutaneous autoimmune manifestations as potential indicators of graft rejection and underscores the need to investigate shared immunopathogenic pathways, including the contribution of non-HLA antibodies in the context of antibody-mediated rejection.

### Patient perspective

The rash developed shortly after I retrieved a ball from the backyard fence, leading me to suspect exposure to poison sumac in the area initially. However, the blisters progressively increased in size, spread to multiple areas of the body, and became increasingly painful and itchy. Following an extensive diagnostic evaluation, a skin biopsy confirmed the presence of a rare dermatologic condition. My skin and lung disease worsened concurrently, and I felt that the progression of my skin condition contributed to the deterioration of my pulmonary symptoms. I was started on therapy for antibody-mediated rejection, and I was more concerned about the potential side effects of the treatment than the rejection itself. Since beginning the allergy immunotherapy, the itching associated with my skin disease has improved; however, I continue to struggle with my lung disease, mostly with the significant chest congestion with copious respiratory secretions.

## Data Availability

The original contributions presented in the study are included in the article/Supplementary Material, further inquiries can be directed to the corresponding author.
